# Author’s Response to Peer Reviews of “The Influence of COVID-19 Vaccination on Daily Cases, Hospitalization, and Death Rate in Tennessee, United States: Case Study”

**DOI:** 10.2196/32459

**Published:** 2021-08-12

**Authors:** Ali Roghani

**Affiliations:** 1 Division of Epidemiology University of Utah Salt Lake City, UT United States

**Keywords:** COVID-19, pandemic, vaccination, vaccine, strategy, vaccination strategy, hospitalization, mortality rates, older adults, mortality

*This is the author’s response to peer-review reports for “The Influence of COVID-19 Vaccination on Daily Cases, Hospitalization, and Death Rate in Tennessee, United States: Case Study.”*

## Round 1 Review

The author of the manuscript [1] is grateful to the editor and reviewers [2,3] for their invaluable input and feedback.

### Anonymous Reviewer [2]

#### Specific Comments

##### Major Comments

1. Thank you for your comment. I have updated the title based on the suggestion.
2. Thank you for your suggestion. I have added arguments and statistics to the Introduction.
3. I have developed a section for measures.
4. Thank you for your recommendation. I have developed a Methods section based on your suggestion.
5. Thank you for your comment; it is an important point. My preliminary analysis showed that the results were fairly consistent up to the first month of vaccination. Additionally, it takes time to see the effectiveness of vaccination. Therefore, this study began on the first day of vaccination.

##### Minor Comments

1. Thank you. I have reviewed the writing and have improved it.
2. I have changed it.
3. I have changed it.
4. Thank you. I have changed the language.

### Anonymous Reviewer [3]

#### Specific Comments

##### Major Comments

1. I have added in a study that discusses another methodology, which was different from the US vaccine policy.
2. Thank you. I have developed a section for measures and statistical analysis.
3. Thank you. I have added a section on implications.

##### Minor Comments

1. I have reviewed the paper’s write-up.
